# Supplementary material for: Biodegradation of Tetrahydrofuran by the Newly Isolated Filamentous Fungus Pseudallescheria boydii ZM01
Source: Microorganisms. 2020 Aug 5;8(8):1190. doi: 10.3390/microorganisms8081190 (PMC7464125; doi:10.3390/microorganisms8081190)
Supplement: Supplementary file 1 [file microorganisms-08-01190-s001.pdf]

microorganisms

Article

Biodegradation of tetrahydrofuran by the newly isolated filamentous fungus *Pseudallescheria boydii* ZM01

Hao Ren, Hanbo Li, Haixia Wang, Hui Huang and Zhenmei Lu *

MOE Laboratory of Biosystem Homeostasis and Protection, College of Life Sciences, Zhejiang University, Hangzhou 310058, Zhejiang, China; 11707034@zju.edu.cn (H.R.); 21707029@zju.edu.cn (H.B.L.); 11207031@zju.edu.cn (H.X.W.); huanghuilengyue@163.com (H.H.)

* Correspondence: lzhenmei@zju.edu.cn

Supplementary data includes 4 figures.

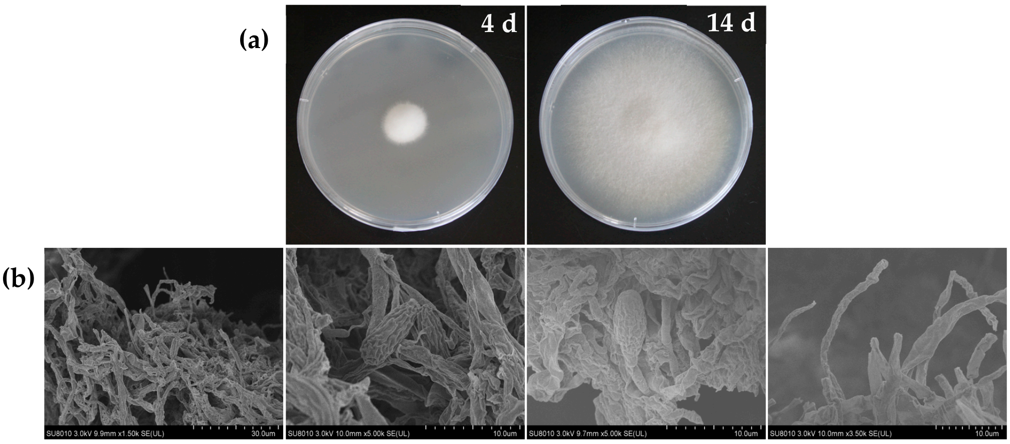

Figure S1. Colony morphology and microscopic morphological characteristics of strain ZM01. (**a**) Colony of strain ZM01 growing on PDA plates after 4 d and 14 d of incubation at 30 ℃. (**b**) The scanning electron microscopic photographs of strain ZM01 after cultivation at 30 ℃ for 14 d.

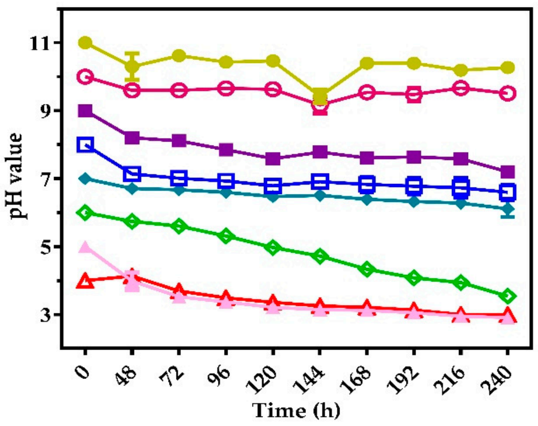

Figure S2. Detection of pH values during THF degradation at different initial pH values by strain ZM01. The symbols indicate initial pH of 4.0 (△), 5.0 (▲), 6.0 (◇), 7.0 (◆), 8.0 (□), 9.0 (■), 10.0 (○), 11.0 (●).

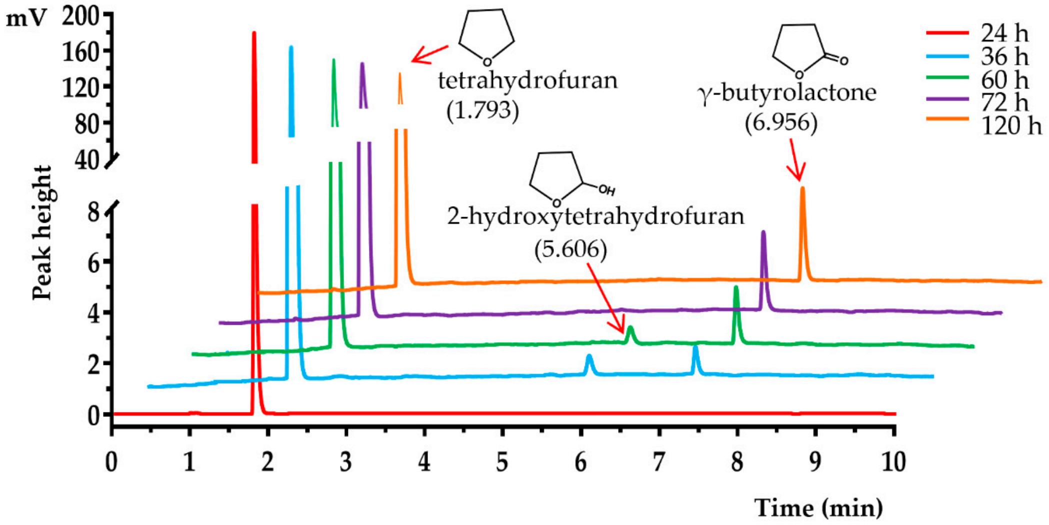

Figure S3. Detection and identification of metabolites in the biodegradation of 50 mM THF by strain ZM01 by GC analysis.

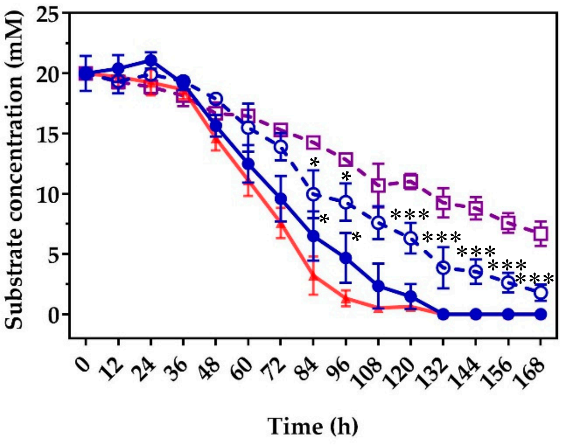

Figure S4. The degradation curve of strain ZM01 using THF and γ-butyrolactone as substrates. The red line represents cultivation with THF as the sole substrate, the purple line represents cultivation with γ-butyrolactone as the sole substrate, and the blue line represents cultivation with THF and γ-butyrolactone as substrates. The solid line represents the THF concentration change, and the dotted line represents the γ-butyrolactone concentration change. Significance was analyzed by Student's *t*-test ($n = 3$): *$p < 0.05$; **$p < 0.01$; ***$p < 0.001$.
